# Treatment of Nævus

**Published:** 1893-03-04

**Authors:** 


### ROYAL INFIRMARY, EDINBURGH.

### Treatment of Nævus.

Operations for the cure of nævi are in most instances undertaken with a view to removing an unpleasant deformity rather than to avert any danger, near or remote, to the patient. This being so, it behoves the surgeon to carefully consider whether the probability of success in any individual case outweighs the risk of his leaving as bad a disfigurement in the shape of a scar, or even of absolute failure. Meddlesome surgery is the more to be avoided inasmuch as fully fifty per cent. of nævi disappear spontaneously. This does not apply to birth marks or port wine stains, which are exceedingly persistent, and, unfortunately, very little amenable to surgical treatment.

Nævi of three varieties present themselves:—

(1) The characteristic *Cutaneous Nœvus*, which is usually situated on the face. Its appearance is well described by the synonym of "port wine stain." The shade varies in different specimens and at different times even in the same specimen. The patch is well defined and level with the surrounding skin. Unless when very small, this form is best left alone, as any attempt at removing it usually fails, and even when moderately successful the resulting scar is no less a deformity than the disease was. In the hands of Mr. John Duncan, whose teaching and practice on the subject of nævus is largely followed in this hospital, fairly satisfactory results have been obtained by repeated painting with liniment of iodine, setting up a degree of dermatitis which has resulted in occlusion of the dilated capillaries.

(2) The *Sub-Cutaneous Nævus* is situated in the tissues underneath the skin, and quite unattached to it. It is exceedingly rare, and its treatment is practically the same as that of the next variety.

(3) The *Mixed Nævus* is chiefly sub-cutaneous, but the dilatation of vessels implicates also the skin, projecting slightly beyond its surface.

In considering the advisability of undertaking active measures several points must be taken into account. (1) The age of the patient. It has already been said that half the cases of nævi recover spontaneously. Not only so, but at certain periods of life, for example, at the first and second dentition and at puberty, they not unfrequently disappear. (2) Is the nævus increasing in size? or (3) Is it causing pressure symptoms, or in danger of giving rise to hæmorrhage? If so, then "cæteris paribus," surgical interference is needed. (4) What amount of disfigurement is it causing? If on a part of the body covered by the clothes obviously it may be left alone, if it is stationary and doing no harm. If on an exposed part the probability of leaving a scar is the only point which arises.

Of operative measures the choice is practically between (1) Electrolysis, and (2) Excision. Other means are sometimes used here; for example, in exceedingly small nævi a drop of strong nitric acid on a pin, or ethylate of sodium painted on the outer edge; but their applicability and success are alike limited.

*Electrolysis* is used for mixed and subcutaneous nævi occurring on exposed parts of the body. It leaves no scar, is safe and successful. The principle is to introduce into the nævus escharotic substances in an extreme state of subdivision, which will immediately act on and coagulate the tissues, so setting up condensation of the swelling. Mr. Duncan uses a Bunsen or Smee's battery of four or six cells. Both poles are introduced into the tumour; at the positive pole oxygen and nitric acid are given off and a firm slough is formed, while at the negative pole hydrogen and caustic potash are evolved, and a larger but softer slough results. The needles are moved about from time to time to set up numerous foci of coagulation, but care must be taken not to come too near the skin lest a slough be formed there. The needles are slowly withdrawn to avoid bleeding, and the punctures covered by collodion and a dressing of dry wool. Two or three applications at intervals of eight or ten weeks may be required to obliterate an ordinary sized nævus.

*Excision*, carried out aseptically, is preferable on covered parts where a scar is immaterial, and where the nævus is not excessively large. By going wide of the tumour bleeding is avoided.

*Ligature* presents almost no advantages over excision, and is less certain in its results.

